# Testing the success of real-time online delivery channel adopted by higher education institutions in the United Arab Emirates during the Covid-19 pandemic

**DOI:** 10.1186/s41239-021-00283-w

**Published:** 2021-09-07

**Authors:** Iffat Sabir Chaudhry, Rene Paquibut, AbuReza Islam, Habib Chabchoub

**Affiliations:** College of Business, Al Ain University, Abu Dhabi, United Arab Emirates

**Keywords:** E-learning system, Information quality, Perceived benefits, Distance learning, Higher education institutions, United Arab Emirates

## Abstract

E-learning was mandated for all the higher education institutions (HEI) in the United Arab Emirates (UAE) to encounter the pandemic and practice social distancing in year 2020. This led education institutions to shift from face-to-face traditional classroom settings to online education channels for delivering education to their students despite being less familiar with the real-time remote learning. The current study attempts to measure the success of e-learning systems adopted by the HEIs in UAE. An e-learning system success measuring the framework based on DeLone and McLean (J Manage Inf Syst 19(4):9–30, 2003) was developed including the measures of quality, system use, perceptual benefits and future outcomes from students’ perspective. A survey was conducted from more than 1200 students studying at different higher education institutions of the UAE region. The findings have implications for educators and policy-makers recommending the success factors of e-learning delivery channels in this region.

## Introduction

Covid-19 has hard-hit the world and changed the outlook of all the industries globally including the educational sector. The education institutions in the United Arab Emirates felt the jolts when social distancing was put in practice by the government to control the pandemic adverse effects in the region. The schools, colleges and universities were mandated to shift to online mode, the mechanism for delivering educational services to its students, which otherwise always followed the conventional approach of face-to-face delivery in traditional class-room settings.

The Ministry of Education took a keen interest in developing online teaching standards and implementing this change in all the regional higher education institutions (HEI) practically, as if in times of war. The HEIs responded to this environmental change in no time and adapted to the current needs of the market by shifting from the classic face-to-face teaching to real-time online teaching; in that an all-out transformation took place in the education system, where teachers continued teaching and assessing the learning outcomes through the online platforms like MS Teams, Zoom, Google Hangouts, to name a few, without wasting a single day of teaching. This mode of teaching was new not only for the students but also for the teachers in the region with quality stigmas attached to the online mode of education.

When real-time online teaching was first introduced in UAE early in the year 2020, the expectation was that the pandemic would be overcome by the summer semester, and the HEIs could roll back to the traditional classroom settings once the new academic year starts in Fall (September 2020). However, in mid 2020, the world got hit further by the second wave of the pandemic which mandated the continuity of online teaching for the UAE education institutions once again, till further notice, keeping in view the government regulations of operating the education systems at 25–30% capacity, and the rest learning from home, in order to counter strike the global pandemic.

Given the dynamics of the ongoing efforts to facilitate learning while coping with the pandemic, it is imperative for the HEIs to determine the success of their real-time online teaching mechanism to make the e-learning system as an effective alternative approach to conventional teaching methods during this period and later. This requires a better understanding of the underlying factors that played an instrumental role in the success of e-learning education services in this region, which is as of now, still naïve in this mode of education. Up to this date, there has been no concerted effort in UAE to identify and assert the relevant determinants of online education services at the tertiary level.

Therefore, to fill the existing gap and provide information to the educators and policy for making informed decisions, the current study empirically validates a multi-dimensional framework based on the DeLone and McLean’s IS success model, for measuring the success of the e-learning system adopted by the HEIs in UAE and assess if the real-time online learning system remained useful in achieving its goals of delivering quality education to the students quarantined at their homes during the pandemic, and if they are satisfied with the quality of the system and the information imparted. It further explores the role of the importance of the e-learning system implementation in the wake of the pandemic and user’s prior experience in determining his/her perception of its usefulness and intention to continue with online mode of teaching as an alternative to face-to-face teaching post-Covid19 as well. The findings will answer the following research questions:

1. Did the students find the e-learning system and the information delivered through it useful for achieving course learning outcomes during the pandemic period? Are they satisfied with the quality of e-learning system and the information imparted?
2. Will the e-learning system’s importance and users prior experience of it, play a role in altering students’ satisfaction level and their perception of the systems usefulness?
3. Will students’ satisfaction and positive perception about the e-learning system influence their intention to adopt online mode of teaching on a voluntary basis in the future once the social distancing is removed and face to face teaching is resumed in higher education institutions in UAE?

## Literature review

Communications technology changes how people work and relate to each other. Sociotechnical integration assumes that social and technical systems optimize organizational production (Shockley-Zalabak, 2014). Education institutions were forced by the Covid-19 pandemic to quickly integrate people and technology as the ‘new normal’. Alsabawy et al. (2011) pointed to e-learning as the main outcome of adopting and using the new and more advanced information technology in the education sector. But what constitutes a success in e-learning? According to Dorobat et al. (2019) the assessment of the effectiveness of e-learning depends on students’ performance and satisfaction with e-learning and that there are studies that discuss the success of e-learning as an emerging social and technical concept.

According to Lee-Post (2009) and Alsabawy et al. (2011), the success of e-learning initiatives have been assessed by large number of studies, specially pertaining to learning benchmarks, learning styles, learning environment, learning outcomes, teaching practices and cost-benefits. However, there is marginal agreement on the factors that contribute to the success of e-learning. Lee-Post (2009) recognized the DeLone and McLean model’s success in bringing together an integrated view of information systems’ success factors, by instilling a process approach. According to Alsabawy et al. (2011), the DeLone and McLean model is the most important measurement approach in the field of e-learning which is commonly used by the researchers and practitioners. DeLone and McLean were the first to introduce the model of success of an information system in 1992 (ISS model). The six constructs of the ISS model are: system quality, information quality, system use, user satisfaction, individual impact and organizational impact. User satisfaction and system use are the main constructs of Delone and Mclean success model that leads to the comprehension of the overall success of the system (Tariq et al., 2018).

According to Tariq et al. (2018) service quality was found as one of the most significant factors in the past studies that affects intention of the user to use the system which consequently impacts on user satisfaction. Tariq et al. (2018) cited the study of Almarashdeh (2016) showing that all system factors (service quality, system quality, information quality) have a significant association with user satisfaction. The e-learning system success depends on the intention of the user for the use of e-learning services. Also, Lee-Post (2009) point out that satisfaction is a critical success factor to build an enduring relationship

According to Delone and McLean (2003) IS success is a multidimensional and interdependent construct which requires the study of the interrelationships among, or to control for, those dimensions. They reviewed 16 published papers which used their model and found that seven tested the association between “system use” (frequency of use, time of use, number of accesses, usage pattern, and dependency) and “individual impacts” (job performance and decision-making performance) and found the association to be significant in each of the studies. Five studies that tested the direct association between “system quality” (ease-of-use, functionality, reliability, flexibility, data quality, portability, integration, and importance) and “individual impacts” (quality of work environment and job performance) reported statistical significance. Four studies that tested the relationship between “information quality” (accuracy, timeliness, completeness, relevance, and consistency) and “individual impacts” (decision-making performance, job effectiveness, and quality of work) were also found significant.

Delone and McLean (2003) clarified a set of important terms used in their model. For example, “System quality”, in the internet environment, measures the desired characteristics of an e-commerce system (e.g. usability, availability, reliability, adaptability, and response time) which are valued by users. “Information quality” captures the e-commerce content issue, which should be personalized, complete, relevant, easy to understand, and secure. “Service quality”, which refers to the overall support delivered by the service provider, applies regardless of who is delivering it, whether internally or externally. The importance of service quality is far greater that previously considered since users are treated as customers can be lost due to poor support services. “Usage” is measured from a visit to and navigation of a Web site, to retrieval of information and execution of a transaction. Customers’ opinions of the e-commerce system remains the basis for measuring “user satisfaction” and should cover the entire customer experience cycle. “Net benefits” capture the balance of positive and negative impacts of the e-commerce on its stakeholders and are considered the most important success measures.

According to Khan et al. (2021) the increasing acceptance of e-learning resulted to the increased interest in studying students’ perception and expectations. Several studies indicated that most students are satisfied with the mode of learning. However, learners are also affected by a host of factors, i.e. age, gender, computer literacy, and learning styles. High-level of engagement can improve the online learning performance of the students through their high satisfaction, increased motivation, and reduced sense of isolation. The study of Khan et al. (2021) analyzed university students’ perception towards e-learning during the Covid-19 pandemic and found that they prefer e-learning because it provides the liberty to connect from anywhere and engage with their class fellows, instructors and materials, leading to a positive attitude formation towards it.

## E-learning system success measuring framework and hypotheses

**Fig. 1:**
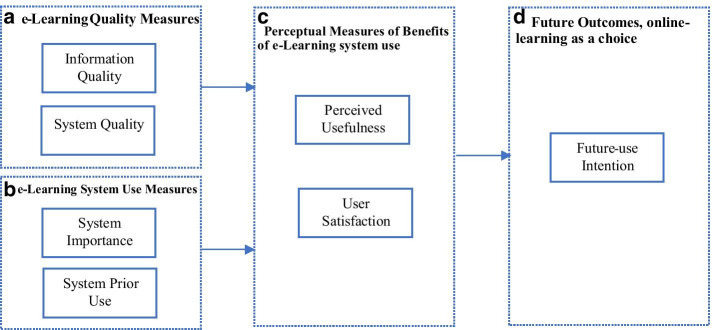
E-learning system success measurement framework

The comprehensive review of the IS success measures based on prior work (DeLone & McLean, 1992, 2003; Seddon, 1997; Wang & Liao, 2008) concluded with the proposed e-learning system success measuring framework (Fig. 1), including the independent measures of ‘system and information quality’ and measures of ‘system importance and system prior use’ to determine the quality and use of the e-learning system adopted by the HEIs; and the dependent measures ‘perceptual usefulness’ and ‘user satisfaction’ to determine the net benefits of the e-learning tools for the students and their ‘future-use intentions’ for e-learning on a volunteer basis, all important measures of e-learning system success (based on DeLone & McLean, 2003; Seddon, 1997 IS success models). This study adopted the ‘system prior-use’ in the context of volunteer usage and the ‘system importance’ in the context of mandatory usage to determine how the previous hands-on experience of e-learning or current mandated implementation of e-learning system can influence systems success. The research model intends to measure the e-learning system’s success from the students’ perspective, therefore, the ‘perceived usefulness’ (Davis, 1989; Seddon & Kiew, 1996) and ‘user satisfaction’ refer to the students’ perceived usefulness of the e-learning system for conducting real-time online classes and their satisfaction with this mode of teaching, respectively.

**Fig. 2:**
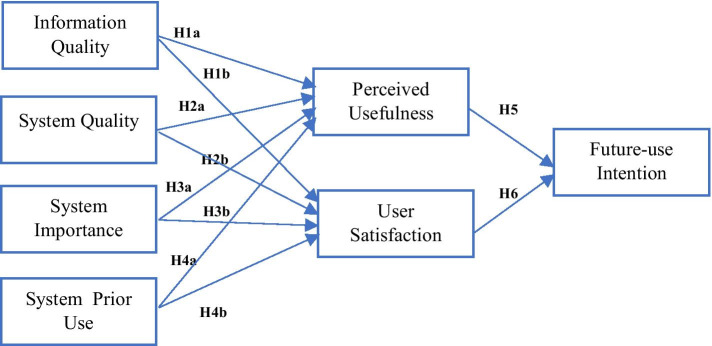
Study research model

The causal interdependencies among the research model (Fig. 2) variables hypothesize that system and information quality along with system importance and its prior use influence the perceived usefulness and satisfaction level of the students, which impact further on their intention to use the e-learning system in the future voluntarily. The constructs of perceived usefulness and user-satisfaction have a dual role in the model, where they are being predicted by the system and information quality, system importance and its prior use and then they predict the future-use intention of the students. The following hypotheses will be tested in the current study:

H1a: Increase in information quality will cause an increase in perceived usefulness.
H1b: Increase in information quality will cause an increase in user satisfaction.
H2a: Increase in system quality will cause an increase in perceived usefulness.
H2b: Increase in system quality will cause an increase in user satisfaction.
H3a: Increase in system importance will cause an increase in perceived usefulness.
H3b: Increase in system importance will cause an increase in user satisfaction.
H4a: Higher system prior use will cause an increase in perceived usefulness.
H4b: Higher system prior use will cause an increase in user satisfaction.
H5: Increase in perceived usefulness will cause an increase in future-use intention.
H6: Increase in user-satisfaction will cause an increase in future-use intention.

## Materials and methods

### Research design

A positivist approach has been adopted to field test the research model. An online survey was conducted to collect the data from the respondents on a self-administered questionnaire.

The study targeted the students enrolled in different universities of the United Arab Emirates to determine their experiences of online learning during the pandemic crisis in the region. The stratified sampling, snowball sampling and convenient sampling techniques were adopted to collect the responses from both public and private sector universities based in different emirates, to keep in view the inclusion of university students across the whole country. The online survey was developed and shared with the prospective respondents via multiple learning platforms as well as social networking sites to reach out the distant participants.

Partial least square structural equation modeling has been adopted to analyze the measurement tools and hypothesized relationships using the multi-stage assessment procedure specified by Ringle et al. (2015) and Hair et al. (2018) using Smart PLS 3 software.

### Measurement models quality assessment

The internal consistency and reliability assessments of all the reflectively measured constructs of the study i.e. InfQual (Doll and Torkzadeh, 1988), SysQual (Doll and Torkzadeh, 1988; Davis, 1989), SysPUse (self-developed), SysImp (Kappelman and McLean, 1991), PerUse (Davis, 1989), UseSat (Seddon and Yip, 1992) and FuInt (self-developed) were undertaken (Chin, 1998) by determining their composite reliability (internal consistency measurement), convergent validity (indicator reliability checking and average variance extraction-AVE), and discriminant validity (following the Forner–Larcker criterion). The detailed results are provided in Table 1a and b.

**Table 1:** a and b Measurement models, quality testing and discriminant validity

(a)					
Constructs	Measurement type	Items	Indicator reliability	AVE	Composite reliability
Exogenous variables					
Information quality	Reflective	Inf_Qual1	0.858	0.741	0.945
		Inf_Qual2	0.846		
		Inf_Qual3	0.891		
		Inf_Qual4	0.846		
		Inf_Qual5	0.850		
		Inf_Qual6	0.872		
System quality	Reflective	Sys_Qual1	0.866	0.717	0.927
		Sys_Qual2	0.806		
		Sys_Qual3	0.840		
		Sys_Qual4	0.877		
		Sys_Qual5	0.843		
System importance	Reflective	Sys_Imp1	0.945	0.889	0.941
		Sys_Imp2	0.940		
System prior-use	Reflective	Prior_Use1	0.868	0.791	0.919
		Prior_Use2	0.905		
		Prior_Use3	0.895		
Endogenous variables					
Future-use intention	Reflective	Fut_Int1	0.720	0.564	0.707
		Fut_Int2	0.631		
Perceived usefulness	Reflective	Perc_Use1	0.861	0.746	0.954
		Perc_Use2	0.887		
		Perc_Use3	0.868		
		Perc_Use4	0.869		
		Perc_Use5	0.869		
		Perc_Use6	0.886		
		Perc_Use7	0.804		
User satisfaction	Reflective	User_Sat1	0.881	0.781	0.935
		User_Sat2	0.899		
		User_Sat3	0.860		
		User_Sat4	0.895		

(b) Constructs	Future-use intention	Information quality	Perceived usefulness	System importance	System prior-use	System quality	User satisfaction
Future-use intention	0.751						
Information quality	0.695	0.861					
Perceived usefulness	0.696	0.816	0.864				
System importance	0.670	0.759	0.734	0.943			
System prior-use	0.371	0.442	0.431	0.317	0.889		
System quality	0.670	0.758	0.771	0.728	0.435	0.847	
User satisfaction	0.745	0.849	0.838	0.733	0.391	0.770	0.884

The indicator reliability test determined that the outer loadings of all the measures of the study constructs remained above the threshold of 0.70 (Hair et al., 2018) except item 2 of future intention construct (i.e. 0.631). However, it was still retained as its deletion reduced the composite reliability score of the construct.

Internal consistency check depicted that the composite reliability score of all the constructs remained higher than the threshold of 0.70, with minimum score of 0.707 (of future use intention construct).

Convergent validity of all the constructs was confirmed with the AVE score higher than 0.5, confirming that the construct explained more than half of the variance of its indicator.

Discriminant validity was determined by using the Fornell–Larcker criteria (requiring the square root of each constructs AVE score to be greater than its highest correlation with any other construct). The results confirmed the discriminant validity of all the constructs as a square root of each constructs AVE score remained greater than its highest correlation with any other construct (details available in Table 1b).

**Fig. 3:**
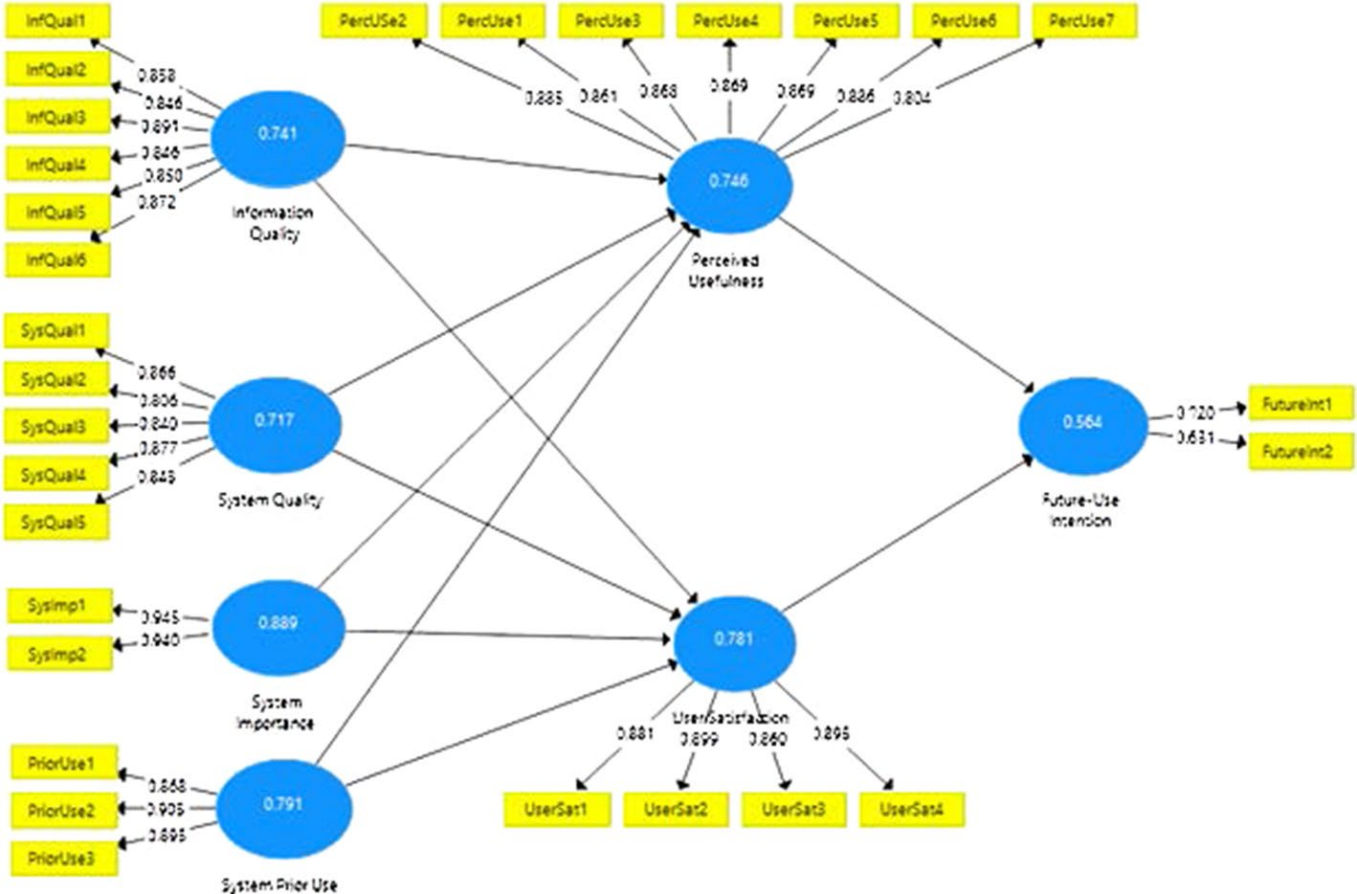
Measurement indicators outer-loadings and AVE

The findings confirmed that the measurement models (Fig. 3) of all the study constructs passed the reliability and internal consistency examination which confirmed their appropriateness for measuring the latent constructs operationalized in the structural model.

### Descriptive data analysis

The survey took 2 weeks in getting the responses from 1351 students studying in 38 public and private universities across UAE. Initially, the issues related to missing data and straight lining, outliers and non-normality were addressed, and the effected data sets were removed accordingly. The filtration process reduced the data sets to 1266 mainly due to missing data. The details of the university-wise students’ participation are shown in Table 2.

**Table 2:** University-wise students’ participation in

Name of the Institution	Emirates	Participants
Al Ain University	Abu Dhabi	511
Higher Colleges of Technology	Majority of UAE Emirates	133
United Arab Emirates University	Abu Dhabi	114
Abu Dhabi University	Abu Dhabi	105
Zayed University	Abu Dhabi and Dubai	95
Khalifa University	Abu Dhabi	20
University of Sharjah	Sharjah	19
IMT University	Dubai	18
City University	Ajman	17
Ajman University	Ajman	14
Curtin University Dubai	Dubai	14
Fatima College of Health Sciences	Abu Dhabi	13
Heroit-Watt University Dubai	Dubai	10
Murdoch University Dubai	Dubai	10
Emirates College of Technology	Abu Dhabi	9
Gulf Medical College	Ajman	8
Emirates Aviation University	Dubai	7
Abu Dhabi Polytechnic	Abu Dhabi	7
Sorbonne University	Abu Dhabi	6
Middlesex University	Dubai	5
Khwarizmi Int'l College	Abu Dhabi	4
Abu Dhabi Vocational Education & Training Ins	Abu Dhabi	3
American University of Ras Al-Khaimah	Ras Al-Khaimah	2
RSCI Institute of Leadership	Dubai	2
New York University	Abu Dhabi	2
Wollongong University Dubai	Dubai	2
Al Dar University College	Dubai	2
Dubai Medical College	Dubai	1
Al Jaheli Institute of Science and Technology	Abu Dhabi	1
Syracuse University in Dubai	Dubai	1
Muhammad V University	Abu Dhabi	1
ADNOC Petroleum Institute	Abu Dhabi	1
Canadian University in Dubai	Dubai	1
Mena College of Management	Dubai	1
American University of Sharjah	Sharjah	1
European Intl College	Abu Dhabi	1
Al Ghurair University	Dubai	1
Dubai University	Dubai	1

The demographic profile of the respondents depicted a wide representation of students belonging to different age groups and gender, enrolled in different years of the undergraduate and post-graduate programs in a variety of fields with different work status. 82% of the participants were Arabic speakers compared to 12% non-Arabic speakers, which depicted the Arab domination in terms of enrollment in the universities’ academic programs compared to those of the expatriate students (see Table 3 for detailed results).

**Table 3:** Students demographic details

Demographic profile	Frequency	Percentage (%)
Gender		
Male	533	41
Female	733	59
Age (years)		
≤ 20	329	26
21 to 30	614	48
31 to 40	287	23
> 40	35	3
Degree program		
Business & Social sciences	659	53
Information Technology	160	13
Applied Sciences	61	5
Engineering	200	16
Arts & Humanities	82	7
Medical studies	65	5
Others	38	3
Year of study		
Grad-year1	289	23
Grad-year2	237	19
Grad-year3	277	22
Grad-year4	284	22
PostGrad-year1	86	7
PostGrad-year2	70	6
Language		
Arabic speaker	1110	88
Non-Arabic speaker	150	12
Work status		
Working	573	45
Not working	689	55

**Fig. 4:**
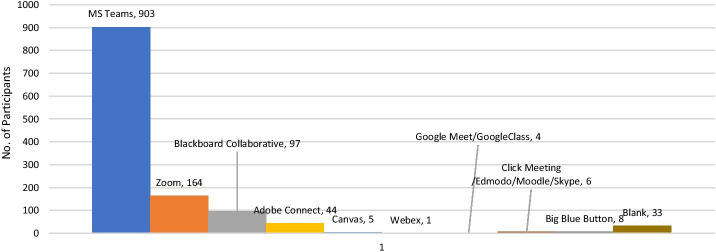
E-learning platforms adopted by the U.A.E. based higher educational institutions

It was identified that the universities across the country adopted different online learning platforms (Fig. 4), including MS Teams, Zoom, Blackboard Collaborative, Adobe Connect, Canvas, Webex, Google meet, Google Classroom, Click meeting, Edmodo, Moodle, Big Blue Button and so on, for coordinating and teaching purposes. However, out of every 10 students who participated in the study, 7 were using MS Teams in their universities as an online learning platform, followed by Zoom (used by 13% of participants) and Blackboard collaborative (7.6%).

The mean value of all the items measuring InfQual, SysQual, SysPUse, SysImp, PerUse, UseSat and FuInt remained higher than the mid-point value of 2.5, depicting that the students found the quality of e-learning system and the information provided, met up to the standards. They also understood the importance of e-learning systems during the pandemic situation by balancing the work-life of the working students (45% of the study sample), and further perceiving the usefulness for distant learning purposes. The mean values also confirmed that e-learning systems were already in use voluntarily by universities for coordinating, teaching and sharing the course materials with students. The skewness and kurtosis values within the acceptable ranges (i.e. skewness: − 1 to + 1 and Kurtosis: − 2 to + 2) depicted the normality of the data (given in Table 4).

**Table 4:** Means, standard deviation, skewness and kurtosis

Items (Min:1–Max:5)	Mean	Std. deviation	Skewness	Kurtosis
IQ_1_ (..) information that is needed	3.232	1.316	− 0.545	− 0.763
IQ_2_(..) required information on time	3.362	1.302	− 0.653	− 0.575
IQ_3_(..) clear course-related information	3.227	1.328	− 0.547	− 0.813
IQ_4_(..) sufficient course-related information	3.355	1.180	− 0.660	− 0.175
IQ_5_(..) up-to-date course-related information	3.442	1.287	− 0.755	− 0.413
IQ_6_(..) useful format	3.295	1.273	− 0.613	− 0.558
SQ_1_(..) easy to use	3.402	1.326	− 0.707	− 0.594
SQ_2_(..) user friendly	3.568	1.187	− 0.938	0.188
SQ_3_(..) easy to learn	3.360	1.249	− 0.606	− 0.436
SQ_4_(..) do what I want it to do	3.285	1.301	− 0.578	− 0.665
SQ_5_(..) easy to become skillful	3.354	1.348	− 0.608	− 0.734
SI_1_(..) in current pandemic requiring social distancing	3.425	1.335	− 0.632	− 0.632
SI_2_(..) to balance work and personal responsibilities	3.458	1.318	− 0.643	− 0.572
SPU_1_(..) for delivering lectures previously	3.101	1.318	− 0.136	− 1.053
SPU_2_(..) for providing course related info. previously	3.144	1.215	− 0.204	− 0.789
SPU_3_(..) for coordinating with students previously	3.090	1.239	− 0.141	− 0.852
PU_1_(..) understand the course material better	2.897	1.383	− 0.211	− 1.217
PU_2_(..) achieve the course learning objectives quickly	2.981	1.349	− 0.309	− 1.084
PU_3_(..) improves my performance in exams	2.936	1.383	− 0.248	− 1.210
PU_4_(..) makes learning process flexible	3.203	1.349	− 0.499	− 0.896
PU_5_(..) makes learning interactive	3.064	1.351	− 0.384	− 1.304
PU_6_(..) makes learning easier	3.063	1.394	− 0.361	− 1.136
PU_7_(..) balance my education and work/personal life	3.266	1.340	− 0.516	− 0.806
US_1_(..) helps to meet the learning outcomes	3.216	1.311	− 0.555	− 0.771
US_2_(..) makes more productive use of resources	3.133	1.330	− 0.459	− 0.914
US_3_(..) perfect replacement for in-class learning	2.905	1.456	− 0.199	− 1.391
US_4_(..) satisfied with the e-learning system	3.130	1.372	− 0.419	− 1.017

## Testing and results

**Fig. 5:**
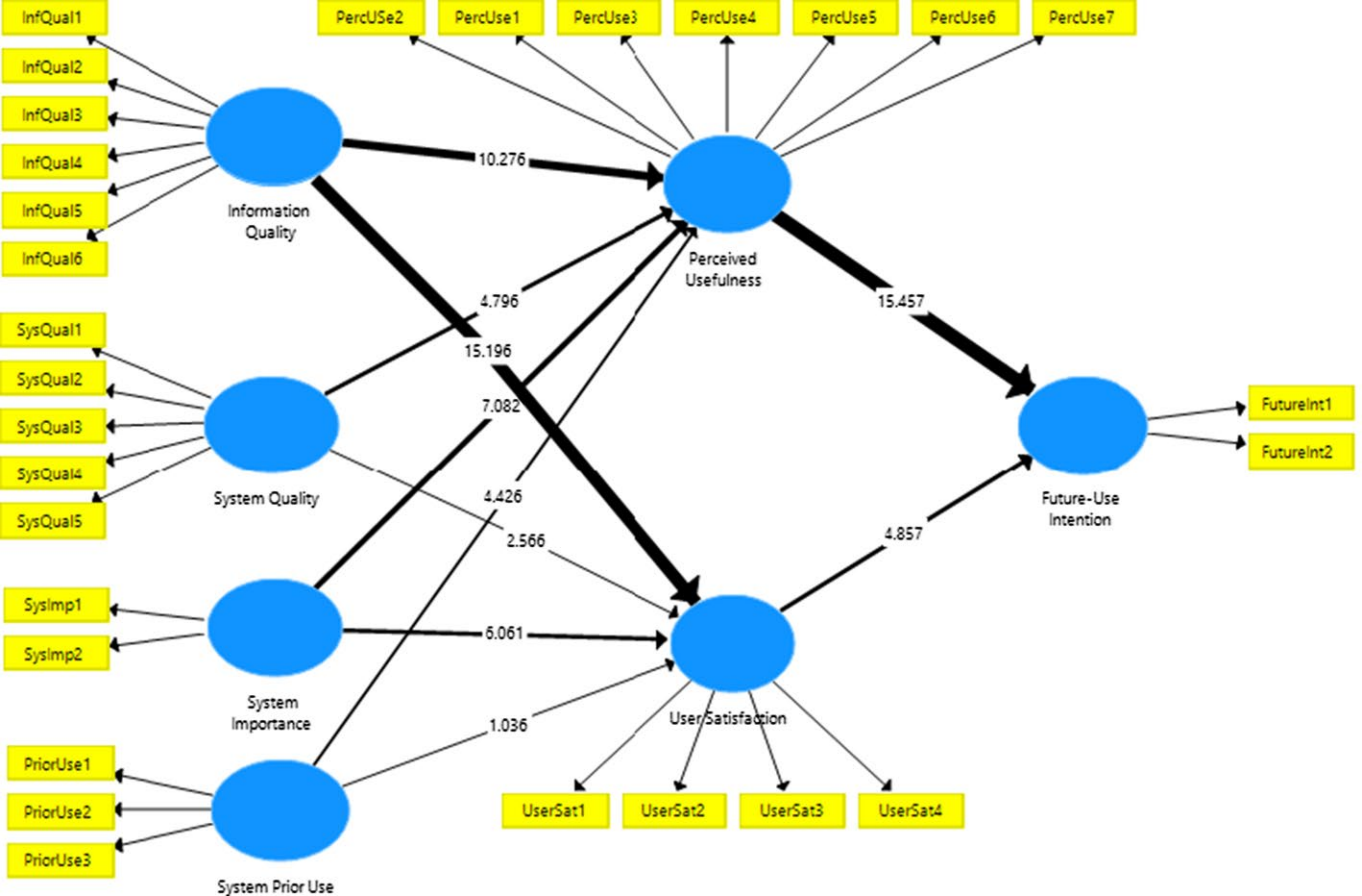
Structural path T-values estimation

The research model’s predictive relevance and the hypothesized relationship of the constructs (Fig. 5) are examined in this section encompassing the testing of collinearity, path significance, R^2^ and *f*^2^ effect size based on the recommendations of Henseler and his colleagues (2009).

### Multicollinearity assessment

The multicollinearity assessment of InfQual, SysQual, SysPUse and SysImp was conducted (using IBM SPSS) as first set of constructs predicting the perceived usefulness and user-satisfaction level. The results indicated that the predictor constructs (InfQual, SysQual, SysPUse and SysImp) had the VIF values less than 5 and the tolerance level higher than 0.2. Next, the multi collinearity assessment of PerUse and UseSat was conducted being predictors of student’s intention of using online learning systems in the future. The results confirmed that both the constructs were free from multicollinearity as they met the thresholds of VIF (less than 4.0) and tolerance level (greater than 0.2).

### In-group variances

A series of non-parametric tests were run to determine any significant differences among the respondents based on age, gender, work status, degree program and year of study to understand the data from different dimensions before moving to the hypotheses testing.

Mann–Whitney U test results (Table 5) confirmed a significant difference between the overall satisfactions of the working versus non-working students with the e-learning system (p-value < 0.001). Working students had a higher level of satisfaction with the e-learning experience compared to non-working students. This result can be attributed to the accessibility of lectures and materials from wherever required instead of driving to the campus all the way to attend the class. Also, they perceived e-learning systems useful for balancing their work and personal life due to which they had a higher intention to continue with e-learning in the future as when traditional classroom teaching would roll back in pandemic free environment. The R values confirmed that the effect of difference between the working and non-working students with respect to their overall satisfaction with the system (r = 0.15), perception about its usefulness for balancing work life (r = 0.14), and intention to use it in the future (r = 0.16), all were meaningful (Cohen, 1988).

**Table 5:** Mann–Whitney U test results

Ingroup variances	Overall user satisfaction	Perceived usefulness for work-life balance	Future-use intention
Working	n = 568 Mean = 679.61 Median = 4.000	n = 566 Mean = 678.19 Median = 4.000	n = 569 Mean = 687.59 Median = 4.000
Non-working	n = 687 Mean = 576.49 Median = 3.000	n = 683 Mean = 580.92 Median = 3.000	n = 685 Mean = 577.58 Median = 3.000
Mann–Whitney U	160,680.500	163,184.500	160,690.500
Z	− 5.221	− 4.914	− 5.552
p-value	0.000	0.000	0.000
R (effect size)	0.15	0.14	0.16

An interesting difference was observed among the students registering in different years of the program. The findings of Kruskal–Wallis test (given in Table 6) highlighted that the differences in the overall satisfaction level of the students studying in the post-graduate programs were significant compared to undergraduate students (p-value < 0.001). Additionally, the students enrolled in senior years of undergraduate programs and post-graduate programs perceived e-learning uses differently compared to those of the freshmen (p-value < 0.001). These findings can be attributed to the professional engagements of the senior graduate and master-level students. An introduction to e-learning helped them to balance their work/life with their educational endeavors through the ease of accessibility to the online lectures (while sitting in offices or at home) and flexibility of learning at their discretion (with supplemental recorded lectures). The study found the influence of the in-group variances in satisfaction and perception of the students on their varied intentions of continuing with online mode of learning on a volunteer basis (p-value < 0.05).

**Table 6:** Kruskal–Wallis H test results

Ingroup variances	Overall user satisfaction	Perceived usefulness for work-life balance	Future-use intention
Bachelors Y1	n = 289 Mean = 526.58 Median = 3.000	n = 288 Mean = 557.38 Median = 3.000	n = 288 Mean = 562.20 Median = 3.000
Bachelors Y2	n = 237 Mean = 588.42 Median = 3.000	n = 237 Mean = 580.34 Median = 3.000	n = 235 Mean = 601.90 Median = 3.000
Bachelors Y3	n = 277 Mean = 650.18 Median = 3.000	n = 277 Mean = 637.66 Median = 4.000	n = 277 Mean = 655.46 Median = 3.000
Bachelors Y4	n = 283 Mean = 627.38 Median = 3.000	n = 284 Mean = 621.06 Median = 4.000	n = 284 Mean = 627.60 Median = 3.000
Masters Y1	n = 86 Mean = 696.62 Median = 4.000	n = 86 Mean = 708.95 Median = 4.000	n = 86 Mean = 616.63 Median = 3.000
Masters Y2	n = 70 Mean = 766.34 Median = 4.000	n = 70 Mean = 757.22 Median = 4.000	n = 70 Mean = 692.74 Median = 3.500
Chi-square	42.219	30.244	14.421
p-value	0.000	0.000	0.013

No significant difference was observed within the group based on degree programs. This finding can be the result of extra step taken by the universities, where students registered in degree programs with practical labs (e.g. engineering, pharmacy, medical and so on) were also attending few classes (face-to-face) assigned on campus (divided into cohorts with less than 10 students in each meeting with negative PCR test results) along with e-classes, to accommodate their practical training. Subsequently, the hypotheses of the study were tested. The results are described below.

### Hypotheses testing

The bootstrapping procedure was run to determine the path coefficient significance of hypothesized relationships between the constructs (see Table 7 for detailed results).

**Table 7:** Hypotheses testing results

#	Study hypothesis	T-statistics	p-values	Status
H-1a	Information quality → Perceived usefulness	10.276	0.000***	Accepted
H-1b	Information quality → User satisfaction	15.196	0.000***	Accepted
H-2a	System quality → Perceived usefulness	4.796	0.000***	Accepted
H-2b	System quality → User satisfaction	2.566	0.010**	Accepted
H-3a	System importance → Perceived usefulness	7.082	0.000***	Accepted
H-3b	System importance → User satisfaction	6.061	0.000***	Accepted
H-4a	System prior-use → Perceived usefulness	4.426	0.000***	Accepted
H-4b	System prior-use → user satisfaction	1.036	0.300	Rejected
H-5	Perceived usefulness → Future-use intention	15.457	0.000***	Accepted
H-6	User-satisfaction → Future-use intention	4.857	0.000***	Accepted

Total indirect effects	T statistics	p values
Information quality → Future-use intention	11.625	0.000***
System quality → Future-use intention	4.532	0.000***
System importance → Future-use intention	7.151	0.000***
System prior-use → Future-use intention	3.892	0.000***

Specific indirect effects	T statistics	p values
Information quality → Perceived usefulness → Future-use intention	8.917	0.000***
System quality → Perceived usefulness → Future-use intention	4.556	0.000***
System importance → Perceived usefulness → Future-use intention	6.185	0.000***
System prior-use → Perceived usefulness → Future-use intention	4.272	0.000***
Information quality → User satisfaction → Future-use intention	4.699	0.000***
System quality → User satisfaction → Future-use intention	2.194	0.028*
System importance → User satisfaction → Future-use intention	3.685	0.000***
System prior-use → User satisfaction → Future-use intention	0.977	0.328

Note: *Significant at.05; **Significant at .01; ***Significant at 0.001

Hypotheses 1a and 1b were tested to determine if high information quality increased the perceived usefulness of the e-learning system (H-1a) and also if it increased the students’ satisfaction with the online learning mechanism (H1b). The t-values confirmed that good quality information played as catalyst to increase the user satisfaction (t-value = 15.196) as well as a significant positive perception of the system’s usefulness (t-value 10.276) at 1% level of significance (p-value < 0.001). The findings accepted hypotheses 1a and 1b.

Next, hypotheses 2a and 2b were tested to assess if high quality of e-learning system impacted on user’s perception regarding the usefulness of the e-learning system (H2a) and his/her satisfaction level (H2b). The findings confirmed the significant positive impact of e-learning system quality on the user’s perceived usefulness of the online system with t-value of 4.796, significant at 1% (p-value < 0.001) thus, accepting hypothesis 2a. Secondly, the findings also accepted hypothesis 2b depicting the significant impact of e-learning system quality on students’ satisfaction level. The t-value remained 2.566 (p-value = 0.01).

Subsequently, hypotheses 3a and 3b were tested to determine if the importance of the utilization of the e-learning system had any impact on the user’s perception of the online learning system usefulness and his/her satisfaction with the online learning. The t-statistics results confirmed a significant positive relationship between the importance of system utilization and its’ perceived usefulness (t-value = 7.082) and users’ satisfaction level (t-value = 6.061), thus accepting the hypotheses 3a and 3b. The t-values for both the hypotheses remained significant at 1%.

Afterwards, hypothesis 4a and 4b were tested to evaluate if students’ prior experience (pre-pandemic) of e-learning system usage for course administration and coordination on a voluntary basis, influenced the user’s perception regarding the online learning usefulness and their satisfaction with the system. The findings confirmed that students who had earlier used e-learning system as part of the learning process perceived the online teaching system useful (t-value = 4.426; p-value < 0.001); this demonstrates the acceptance of hypothesis 4a. However, the students prior hands-on experience of online learning system did not influence their satisfaction level (p-value > 0.5), thus rejecting hypothesis 4b.

After testing the influence of information quality, system quality, system importance and system prior-use on students’ perception regarding the system’s usefulness and their satisfaction with the online learning, their subsequent impact was tested on their intentions of using the e-learning system in the future. Hypothesis 5 (i.e. students’ perceived usefulness of the e-learning system will influence their intention to continue using the online learning system in the future as well) was accepted with p-value < 0.001 and t-statistics 15.457; this is followed by hypothesis 6 stating that student’s satisfaction with the e-learning system will impact on their intention to use the online mode of learning in the future as well. The findings accepted hypothesis-6 (p-value < 0.001; t-statistics = 4.857) confirming that once the pandemic is over and mandatory social distancing rules by the UAE government are reverted, students would still be interested in continuing to prefer the online mode of learning to balance their work and life.

The findings showed that the total effect of information quality on students’ satisfaction and students’ perception of system’s usefulness is greater than that of system quality supporting that the quality of the information imparted to the students during online learning played a dominant role in influencing the students’ satisfaction and the perceived benefits of the e-system for achieving their learning outcomes. It was also reported that the quality of information had a strong influence on their intentions to continue with e-learning in the future as well (t-value = 11.625); this is followed by the importance of online teaching system implementation (t-value = 7.151) for personal reasons or to comply with the government regulations in the wake of pandemics or any other national or regional level crisis. The detailed results are provided in Table 7.

Subsequent to hypotheses testing, the predictive relevance of the exogenous constructs of the current study were assessed with R square and f square effect sizes.

### Predictive relevance: R square and *f*^2^ effect size

The R^2^ of the endogenous constructs of PerUse and UserSat were determined to evaluate the predictive strength of the exogenous constructs in the research model. The findings indicated that the adjusted R^2^ of PerUse and UserSat remained 0.711 and 0.741, respectively, stating that more than 70% variance in students’ perception of e-learning system’s usefulness and their satisfaction with it, can be explained by the quality of the information imparted to them through these e-learning tools, quality of the e-learning system used, importance of the implementation of online mode of learning, in response to personal and external requirements, and their prior experiences of these e-learning tools, as part of course administration on a voluntary basis.

PerUse and UseSat had a dual role in the research model where they remained endogenous constructs while being predicted by InfQual, SysQual, SysImp and SysPUse and subsequently, predicting FuInt as exogenous constructs. The R^2^ of FuInt remained 0.642, confirming that at least 64% variance in students’ intention to continue with online learning in future is based on how useful they perceived the online learning system and how satisfied they remained with the online teaching during the pandemic period when face-to-face teaching was abandoned in the higher education institutions as per the directives of the government.

The R^2^ values of 0.711, 0.741 and 0.642 of the endogenous constructs PerUse, UseSat and FuInt, respectively, supported the substantial to moderate level predictive relevance of the exogenous constructs in the current study (Henseler et al., 2009; see Table 8).

**Table 8:** R square values

Endogenous constructs	R square	R square adjusted
Perceived usefulness	0.712	0.711
User satisfaction	0.742	0.741
Future-use intention	0.643	0.642

Further to this, *f*^2^ effect size was calculated to determine the specific contribution of exogenous constructs in students’ perceived usefulness of the system and their satisfaction level and their subsequent influence on their intention to use the e-learning approach in the future as well. Khalilzadeh and Tasci (2017) suggested that the *f* square value of 0.3, 0.15 and 0.1 depict large, moderate and small size effects, respectively, of the predictor on the endogenous variable in management research. As a rule of thumb, *f* square less than 0.02 shows no effect of the independent variable on the dependent variable.

The *f* square results (Table 9) indicated that InfQual (*f*^2^ = 0.15) has moderate effect on user’s perceived usefulness of the e-learning system followed by very small effects of SysImp (*f*^2^ = 0.08) and SysQual (*f*^2^ = 0.028) with no effect of SysPUse (*f*^2^ = 0.018), though the system prior use had a significant positive impact on the user’s perception about the e-learning system with p-value < 0.001. Additionally, InfQual (*f*^2^ = 0.320) has the larger effect on users’ satisfaction with smaller effect of SysImp (*f*^2^ = 0.057). However, SysPUse (*f*^2^ = 0.001) and SysQual (*f*^2^ = 0.009) effect sizes remained insignificant on the users’ satisfaction level. Notwithstanding, the quality of the system had significant positive influence on user’s satisfaction with p-value < 0.01.

**Table 9:** *f* square effect size

Exogenous constructs	Perceived usefulness	User satisfaction	Future-use intention
Information quality	**0.148**	**0.320**	
System importance	0.080	0.057	
System prior use	0.018	0.001	
System quality	0.028	0.009	
Perceived usefulness			**0.246**
User satisfaction			0.026

Significant results are bolded

Next, the *f* square values showed a moderate effect of PerUse (*f*^2^ = 0.246) and a minor effect of UseSat (*f*^2^ = 0.026) on the students’ future intentions of choosing e-learning over the traditional classroom mode of learning. The results are discussed in the following section.

## Discussion and conclusion

The current study empirically assessed the success of the e-learning system adopted by the higher education institutions in UAE during the pandemic to deliver real-time online education to the students while staying at home. The e-learning success measuring model used in this study was based on DeLone and McLean (2003) and Seddon (1997) work on information systems success model, including the measures of: information quality, system quality, system importance, system prior-use, perceived usefulness, user satisfaction and future-use intent.

The preliminary findings confirmed that students across the UAE tertiary education institutions experienced the above average information quality and system quality (with mean values of all the items above the mid-point of 2.5) when shifting to the online education delivery channel from the conventional face-to-face classroom teaching to encounter the pandemic. Also, the students realized the importance of the remote learning and e-learning system implementation in the wake of pandemic emergency and personal pressures (mean values above 3.4) which inspired them with higher inclination towards the acceptance of the newly introduced real-time online learning by their educational institutions. It is worth mentioning that most of the students (mean values above 3.0) reported that they were already using e-learning platforms in their respective universities for the purpose of sharing course materials, coordination with instructor and learning. This qualifies that e-learning platforms are not new to the students and they already had hands-on experiences which made this entire transition process to the new mode of education delivery channel smooth and successful.

The hypotheses results confirmed that the measures of DeLone and MsLean’s model of IS success were valid measures of e-learning system success supporting the findings of Lee-Post (2009) and Alsabawy et al. (2011). All the hypothesized relationships between the study constructs of information quality, system quality, system importance, system prior-use, perceived usefulness, user satisfaction and future-use intention were accepted except the relationship between the system prior-use and user satisfaction depicting that earlier volunteered-use of the e-learning system for sharing course material or coordinating purposes do not necessarily result in increased satisfaction of the students with the e-learning system adopted for real-time online teaching during the pandemic.

The significant positive relationships of the information quality and system quality with students satisfaction and their positive perception regarding the usefulness of the e-learning system and their intention to continue using the e-learning system in the future (as well), consolidated the significant role of information and system quality in the formation of user satisfaction, positive perception, and future-use intention, all supporting strongly the prior findings of Tariq et al. (2018) and Almarashdeh (2016). The university management needs to focus on delivering quality information to the students and making their systems user-friendly, if they would like the students perceiving the e-learning systems useful and evaluate them satisfactorily for using it in the future as well on a voluntary basis. However, out of the above two, information quality was found to have the strongest direct and total indirect effect on the students’ satisfaction level with the new teaching system, their perceived benefits from the adoption and their intent to continue using it even when it is not mandated by the institutions and government; this indicated its strongest role in developing the positive psychological and behavioral processes of its users. All these suggest that the education institutions, therefore, must prioritize the quality of information and education imparted to the students upon expensive e-learning systems installations, to promote the perceived benefits of the online learning, which is comparatively new to the region. This particular finding is also in line with prior research of Wang and Liao (2008) on the leading role of information quality in e-government system success, validating our research results. However, it is also important to determine in future studies the significance of information quality for e-learning in asynchronous learning environment, when there is no real-time teaching involved and instant feedbacks and immediate responses are not available (Littlefield, 2018), as it was not included in the current study objectives.

The importance of “system use” due to personal and external pressures (pandemic) was found to have a strong impact on students’ psychological and behavioral intents, indicating that when students understand the importance of adopting remote learning tools to deal with current circumstances, they perceive the e-learning system to be useful, and are satisfied with its adoption for achieving the course learning outcomes. Given this impetus, students showed an affirmation and inclination towards continuing with online education delivery, to deal with personal and national emergencies, if they happen to prolong.

The study findings depict the positive attitude of the Emirati students towards the acceptance of online mode of education, which is not yet offered by the universities and colleges in United Arab Emirates except for very few. Most of the higher education institutions adopt e-learning systems to provide educators and learners with personalized learning environments, giving access to resources, materials, and exams and others, but real-time lectures. The students with prior experience of using the e-learning system for accessing materials and taking exams, perceived the e-learning system useful, however, this prior experience of e-learning did not show any impact on their satisfaction with the real-time online learning or intention to use it in future. This means, if universities and institutions look forward to higher satisfaction level of their students with e-learning systems and intend to orient them towards this mode of education in the future (as well), they would need to focus primarily on quality of information provided to them by using the online teaching platforms; this has to be followed by the need to share the importance of adopting the online learning system. Next in line there is a need to focus on system quality and its prior-use as they have significant impact on users’ satisfaction, particularly their perception about the e-learning benefits and future intent, after information quality and systems importance.

The study results further showed that the students who perceived the e-learning systems useful and are satisfied in achieving the course learning outcomes using the online mode of education delivery, intended to continue using the online mode of education (as delivered currently) post-pandemic voluntarily, when traditional classroom teaching will resume. Specially, the working students (i.e. 45% of the study sample of 1267 students) reported higher satisfaction with the e-learning system and also, they perceived it to be very useful for managing their work-life balance. Also, working students showed higher intention of continuing with online mode of education post-pandemic which were attributed to the convenience and easy access to the real-time lectures, as reported by Khan et al. (2021). However, no significant difference was found between the male and female students, in terms of their perceptual assessment and behavioral intent towards e-learning. It would be interesting to explore further in future studies if e-learning will be able to make a ground along with traditional face-to-face learning in Emirates higher education institutions, which otherwise had little acceptance by the educational industry and the government agencies pre-pandemic.

The R-square results confirmed the well-fit regression model with the e-learning system success measures of information quality, system quality, system importance and system prior-use explaining more than 70% variation in users satisfaction with e-learning systems and their perception about its usefulness. Subsequently, user-satisfaction and their perceived benefits explained a 64% variation in students’ intention to continue using the real-time online mode of education in the future, when the pandemic will be controlled, and traditional classroom learning will resume.

The above study findings have several important implications for researchers, educators, institutional administrators and government policy-makers alike. It has provided valid measures for determining the success of e-learning systems adopted by the universities to reduce the exposure of students to the risk of Covid-19. The institutional administrators at the tertiary level of education can enhance their understanding of the priorities of their students that are useful for taking necessary actions to develop their positive attitude and behavior towards the newly adopted e-learning approach. On the other hand, the researchers can develop the validated model further, by including more e-learning system success measures based on the existing theories and empirical studies and make the comparative assessments of the current findings with those of the other countries. This will help in developing the merits with general acceptability of the success measures’ selection, otherwise the findings might be culturally constrained. Primarily, being high power-distance oriented society (Hofstede Insights, 2021), the government authorities and institutional managers in Emirates are highly respected and not questioned. This cultural value might be a corner stone for whole-hearted acceptance of e-learning by educators and students on a large scale over a night. Therefore, the future studies need to determine if the current findings are applicable to the societies with low power-distance value.

The current study was cross-sectional in which responses were gathered from the participants online, after one semester since the introduction of real-time online learning. The online availability of the survey form enabled the investigators to collect the responses from the students enrolled in different states other than the capital city (where study was conducted). In few instances, the participation of the students from each institution was as low as one. However, every single participation from any university/institution was utilized in the analyses, to increase the generalizability of results in UAE educational industry. In future, significant participation of students from each institution must be engaged to determine institution specific e-learning success rate, which was not included in the current study framework. Also, the longitudinal studies in future may help improve our understanding of the factors contributing to the e-learning success over the period of time. Moreover, the study reported the students perspective of e-learning system benefits and their intent to adopt the online learning in the future, however, it is important to take into consideration the perspective of instructors to understand the factors contributing to the better dissemination of online education, identifying simultaneously the constraints and challenges (both internal and external) faced by the faculty members in delivering real-time lectures and administrating the course online. It is also worthwhile to conduct future research on the role of government bodies in making the e-learning mode of education successful in different countries with varied level of socio-economic standings and e-learning development and their adoption.

## Data Availability

The datasets used for the current study are available from the corresponding author on request.
